# Cloning and Functional Characterization of a Lycopene β-Cyclase from Macrophytic Red Alga *Bangia fuscopurpurea*

**DOI:** 10.3390/md15040116

**Published:** 2017-04-11

**Authors:** Tian-Jun Cao, Xing-Qi Huang, Yuan-Yuan Qu, Zhong Zhuang, Yin-Yin Deng, Shan Lu

**Affiliations:** 1 State Key Laboratory of Pharmaceutical Biotechnology, School of Life Sciences, Nanjing University, Nanjing 210023, China; ctjsw@126.com (T.-J.C.); hl_david@msn.com (X.-Q.H.); someoneqyy@hotmail.com (Y.-Y.Q.); njcs2006@126.com (Z.Z.); 2 Jiangsu Institute of Oceanology and Marine Fisheries, Nantong 226007, China

**Keywords:** *Bangia fuscopurpurea*, red algae, lycopene cyclase, carotenoid, metabolism

## Abstract

Lycopene cyclases cyclize the open ends of acyclic lycopene (ψ,ψ-carotene) into β- or ε-ionone rings in the crucial bifurcation step of carotenoid biosynthesis. Among all carotenoid constituents, β-carotene (β,β-carotene) is found in all photosynthetic organisms, except for purple bacteria and heliobacteria, suggesting a ubiquitous distribution of lycopene β-cyclase activity in these organisms. In this work, we isolated a gene (*BfLCYB*) encoding a lycopene β-cyclase from *Bangia fuscopurpurea*, a red alga that is considered to be one of the primitive multicellular eukaryotic photosynthetic organisms and accumulates carotenoid constituents with both β- and ε-rings, including β-carotene, zeaxanthin, α-carotene (β,ε-carotene) and lutein. Functional complementation in *Escherichia coli* demonstrated that BfLCYB is able to catalyze cyclization of lycopene into monocyclic γ-carotene (β,ψ-carotene) and bicyclic β-carotene, and cyclization of the open end of monocyclic δ-carotene (ε,ψ-carotene) to produce α-carotene. No ε-cyclization activity was identified for BfLCYB. Sequence comparison showed that BfLCYB shares conserved domains with other functionally characterized lycopene cyclases from different organisms and belongs to a group of ancient lycopene cyclases. Although *B. fuscopurpurea* also synthesizes α-carotene and lutein, its enzyme-catalyzing ε-cyclization is still unknown.

## 1. Introduction

Carotenoids are one of the largest groups of natural pigments in land plants, algae, bacteria including cyanobacteria and photosynthetic bacteria, archaea, fungi and animals [1,2]. More than 750 carotenoids, not including their *cis*- and *trans*- isomers as distinct compounds, have been isolated and characterized from natural sources [3]. Carotenoids not only serve as essential pigments for photosynthesis and photoprotection in photosynthetic organisms, but also comprise many of the yellow, orange and red colors in flowers and fruits that attract pollinators and seed dispersers in higher plants [4]. Abscisic acid and strigolactones are also derivatives of carotenoids [5].

Higher plants generally have very similar leaf carotenoid profiles, with lutein, β-carotene (β,β-carotene), violaxanthin and neoxanthin being the major components. They may also accumulate specific carotenoids, such as capsanthin in pepper fruits [6], β-carotene in carrot tubers [7] and astaxanthin in *Adonis aestivalis* flowers [8]. In the past 20 years, most of the metabolic reactions in the biosynthesis of carotenoids have been deciphered, thanks to the cloning and characterization of genes encoding metabolic enzymes from different organisms [9]. In plants, the plastidic methylerythritol phosphate (MEP) pathway utilizes glyceraldehyde-3-phosphate and pyruvate to synthesize the 5-carbon (C_5_) isopentenyl diphosphate (IPP) and its isomer dimethylallyl diphosphate (DMAPP). A geranylgeranyl diphosphate (GGPP, C_20_) synthase (CrtE) catalyzes the condensation of these two substrates to produce GGPP as a precursor for the biosynthesis of carotenoids and other diterpenoids. Phytoene synthase (CrtB) utilizes two molecules of GGPP to produce phytoene and thus directs the metabolic flux from GGPP into carotenoid metabolic pathway. Phytoene is then converted to acyclic lycopene (ψ,ψ-carotene) by the combination of desaturases CrtP and CrtQ in eukaryotes, but only CrtI in prokaryotes [2,10].

**Figure 1:**
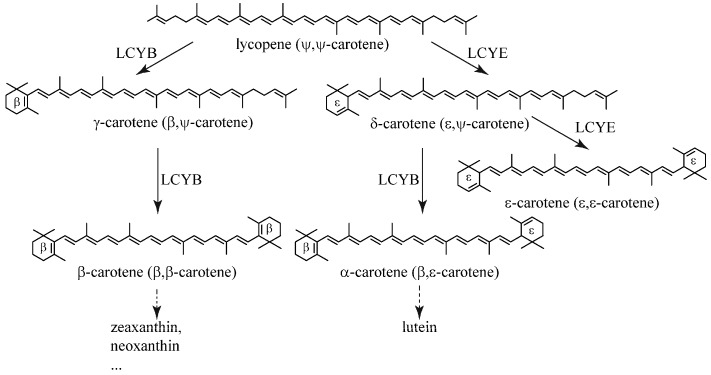
Cyclization of lycopene catalyzed by lycopene β-cyclase (LCYB) and ε-cyclase (LCYE).

Cyclization of lycopene by lycopene cyclases (LCY) is a crucial bifurcation step in the carotenogenic pathway (Figure 1). Each open end of lycopene can be cyclized by lycopene β-cyclase (LCYB) or ε-cyclase (LCYE) to produce β- or ε-ionone rings, respectively (Figure 1). The formation of two β-ionone rings results in the production of β-carotene and its derivatives (e.g., zeaxanthin and neoxanthin), whereas the combination of β- and ε-ionone rings forms α-carotene (β,ε-carotene) and its derivate lutein (Figure 1). Carotenoids with two ε-ionone rings are uncommon in most plants, with lactucaxanthin in lettuce (*Lactuca sativa*) being one of the exceptions [11]. Carotenes with at least one unsubstituted β-ionone ring can be utilized by the human body to produce vitamin A. Therefore, an increase in the activity of LCYB can direct the metabolic flux from lycopene to the production of this group of so-called provitamin A carotenes to increase the nutritional value of crops [12,13].

Cumulative studies on carotenoids in red algae have uncovered broad compositional diversity [2,14,15,16]. Red algae can also be divided into three groups according to their specific carotenoid compositions [15,16]. Although carotenoid constituents with an ε-ionone ring are missing in some primitive species, β-carotene is found in all red algae, suggesting a ubiquitous distribution of the β-cyclization activity in carotenoid biosynthesis [16,17]. *LCYB* of *Cyanidioschyzon merolae* is also the only *LCY* gene from the entire red alga phylum that has been characterized [16,17].

The red algal genus *Bangia* Lyngb., although probably not monophyletic, is among the earliest multicellular eukaryotic photosynthetic organisms [18]. Carotenoids with both β- and ε-rings, including β-carotene, zeaxanthin, α-carotene and lutein, are accumulated in *Bangia* [15,16]. In this work, we isolated a gene encoding an LCY from *Bangia fuscopurpurea*, and functional characterization revealed that this enzyme has only β-cyclization activity.

## 2. Results

### 2.1. BfLCYB Is an Intron-Less LCY Gene

**Figure 2:**
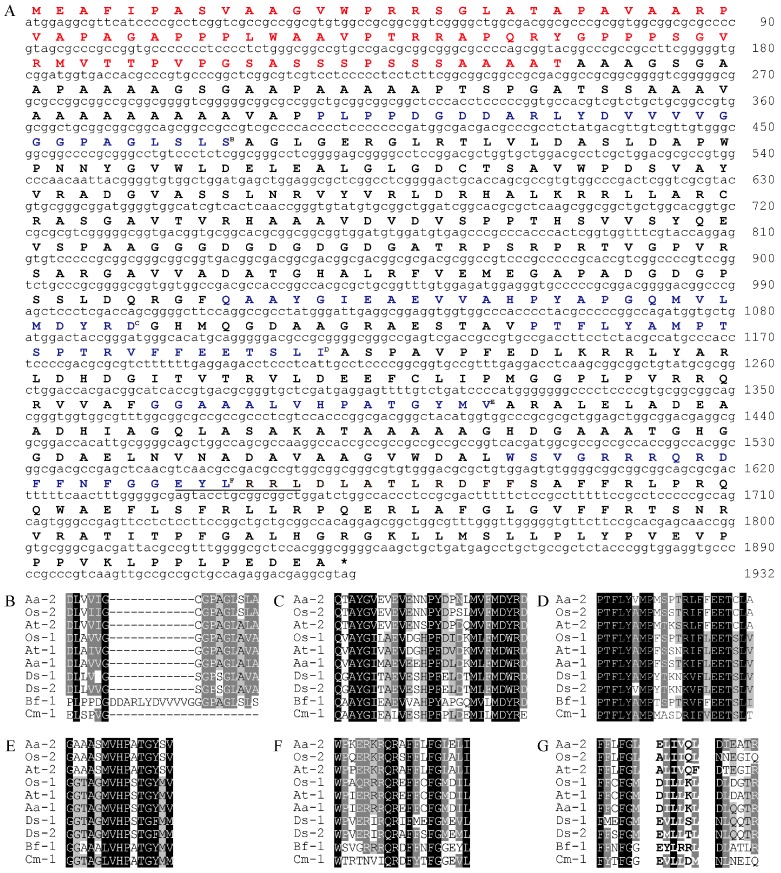
Sequence analysis of BfLCYB. (**A**) *BfLCYB* complementary DNA (cDNA) sequence and its deduced amino acid sequence. Sequence of the predicted N-terminal chloroplast transit peptide is in red. (**B**–**F**) Sequence comparison of the NAD(P)/FAD-binding domain (**B**) and other conserved domains (**C**–**F**). (**G**) Sequence comparison of the six-amino-acid region (in bold) that determines ring numbers of LCYE and flanking regions. The conserved domains in BfLCYB are in blue and indicated by superscript letters, and the ring number-determination region in BfLCYB is underlined in (**A**). Sequences of functionally characterized LCYEs from *Adonis aestivalis* (Aa-2, AAK07430.1), *Oryza sativa* (Os-2, XP_015622198.1) and *Arabidopsis thaliana* (At-2, NP_200513.1) and LCYBs from *A. aestivalis* (Aa-1, AAK07432.1), *O. sativa* (Os-1, XP_015627234.1), *A. thaliana* (At-1, NP_187634.1), *Dunaliella salina* (Ds-1, ACA34344.1; Ds-2, ANY98896.1), *Cyanidioschyzon merolae* (Cm-1, XP_005536481.1) and *Bangia fuscopurpurea* (Bf-1, KX943552) were compared.

From the alignment of functionally characterized LCYs from different organisms, we designed two pairs of degenerate primers. Nested PCR using these primers resulted in amplicons of different sizes. After cloning and sequencing these fragments, a 262 base pair (bp) fragment was found to have high similarity to known LCYs (data not shown). Based on the sequence of this fragment, we successfully amplified a 2218 bp genomic DNA fragment and a 1932 bp complementary DNA (cDNA) encoding a putative protein with 643 amino acid residues, designated BfLCYB in this work (Figure 2A). Sequence alignment demonstrated that *BfLCYB* does not possess any introns. BfLCYB shares 38% sequence identity with the characterized LCYB from *C. merolae* [17]. The sequence of *BfLCYB* has been deposited in GenBank under the accession number KX943552.

Five conserved domains, including an NAD(P)/FAD-binding domain, were previously reported for functionally characterized LCYs [2,10]. These domains were also identified in BfLCYB (Figure 2). However, the NAD(P)/FAD-binding domain in BfLCYB is interrupted by an apparent insertion of 12 additional amino acid residues (Figure 2B). Cunningham and Gantt also identified a six-amino-acid region in LCYE that contributes to the determination of ring numbers in lycopene ε-cyclization products [11]. We found that this region is located immediately downstream from the fifth conserved domain in LCYs (Figure 2F,G). However, in BfLCYB, this region has much lower similarity to other LCYs than either its upstream or downstream flanking regions (Figure 2G).

### 2.2. BfLCYB Localizes in Chloroplasts

**Figure 3:**
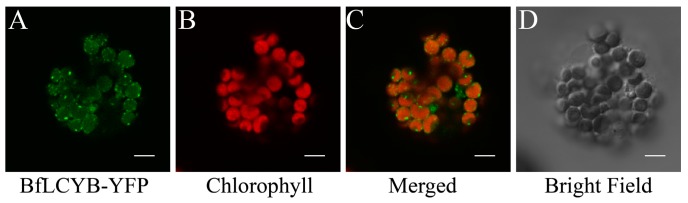
Subcellular localization of BfLCYB. The BfLCYB yellow fusion protein (BfLCYB-YFP) was transiently expressed in *Arabidopsis thaliana* protoplasts, and observed under a confocal microscope. Signals from the fusion protein (**A**) and chlorophyll autofluorescence (**B**) were merged (**C**) for a comparison with the protoplast observed under bright field (**D**). The transient expression experiment was repeated independently five times and only one representative protoplast is shown here. Scale bar, 5 μm.

From online analysis using TargetP, ChloroP and YLoc, BfLCYB was predicted to have a chloroplast localization [19,20,21]. Its N-terminal chloroplast transit peptide predicted by TargetP and ChloroP is in square brackets in Figure 2A. To experimentally confirm its localization, the open reading frame (ORF) of *BfLCYB* was fused in frame to the 5′-end of the yellow fluorescent protein (*YFP*) in pA7-YFP. Due to the lack of a method to stably transform *Bangia*, we transiently expressed the BfLCYB-YFP fusion protein in *Arabidopsis thaliana* leaf protoplasts [22]. The YFP signal of the fusion protein was solely found in chloroplasts and merged nicely with chlorophyll autofluorescence, thus confirming its predicted chloroplast localization (Figure 3).

### 2.3. BfLCYB Is a Bicyclic Lycopene β-Cyclase

**Figure 4:**
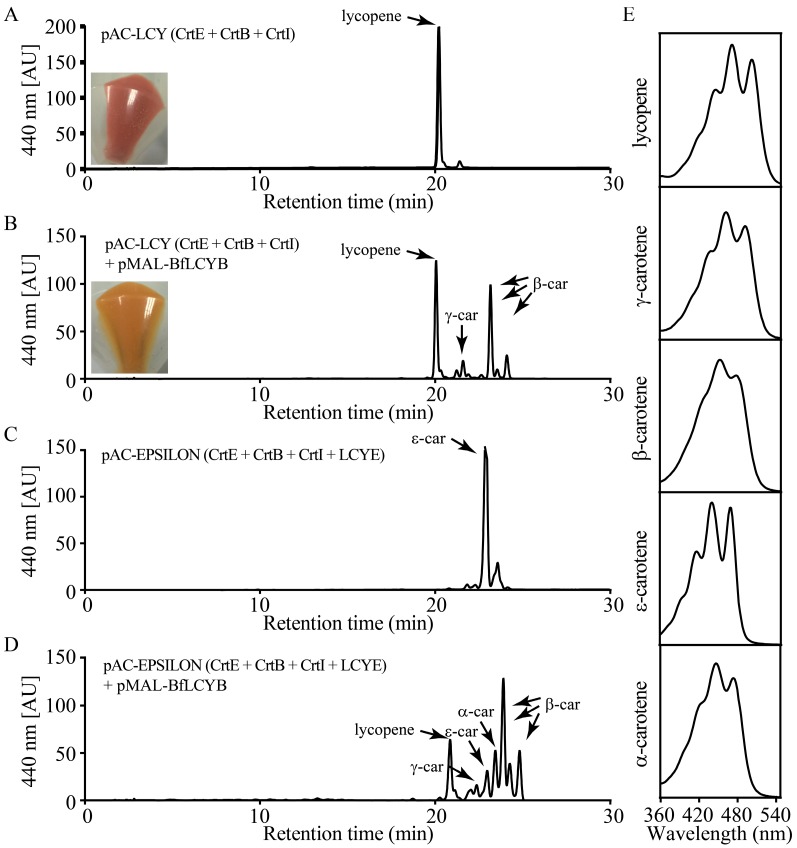
Functional characterization of BfLCYB. (**A**) Production of lycopene in *Escherichia coli* cells transformed with pAC-LCY. (**B**) Production of γ-carotene and β-carotene in *E. coli* cells transformed with both pAC-LCY and pMAL-BfLCY1. (**C**) Production of ε-carotene in *E. coli* cells transformed with pAC-EPSILON. (**D**) Production of α-, ε-, γ- and β-carotene in *E. coli* cells transformed with both pAC-EPSILON and pMAL-BfLCYB. (**E**) UV-visible absorption spectra of different carotenoid products. Insets in (**A**,**B**) showed different colors of the pelleted bacterial cells accumulating lycopene and β-carotene, respectively.

To determine the enzymatic activity of BfLCYB, we used a bacterial pigment complementation system [23]. The plasmid pAC-LCY contains the genes from *Pantoea* for geranylgeranyl diphosphate synthase (*CrtE*), phytoene synthase (*CrtB*) and phytoene desaturase (*CrtI*). *Escherichia coli* transformed with pAC-LCY can utilize endogenous IPP and DMAPP to produce lycopene, which turns the cells pink (Figure 4A). When we co-transformed *E. coli* with both pMAL-BfLCYB and pAC-LCY, the positive transformants harboring both plasmids had an orange color (Figure 4B). High-performance liquid chromatography (HPLC) analysis revealed an accumulation of both the monocyclic γ-carotene (β,ψ-carotene) and the bicyclic β-carotene in these orange cells (Figure 4B). We did not find accumulation of either ε-carotene (ε,ε-carotene) or δ-carotene (ε,ψ-carotene) in the co-transformed bacteria. These results suggested that BfLCYB is able to cyclize one or both ends of lycopene into β-ionone rings but not ε-ionone rings.

We further confirmed the function of BfLCYB by co-transforming pMAL-BfLCYB with pAC-EPSILON. In addition to the genes carried by pAC-LCY, pAC-EPSILON carries the gene from lettuce for LCYE, which catalyzes the ε-cyclization at both ends of lycopene [11]. *E. coli* cells transformed with pAC-EPSILON accumulated ε-carotene, as expected (Figure 4C). When this plasmid was co-transformed with pMAL-BfLCYB, HPLC analysis revealed the accumulation of both α-carotene and β-carotene, together with a significantly reduced level of ε-carotene and a small amount of γ-carotene (Figure 4D). This demonstrated that BfLCYB can also β-cyclize the open end of monocyclic δ-carotene.

### 2.4. BfLCYB Is an Ancient Type of Lycopene Cyclase in Plants

**Figure 5:**
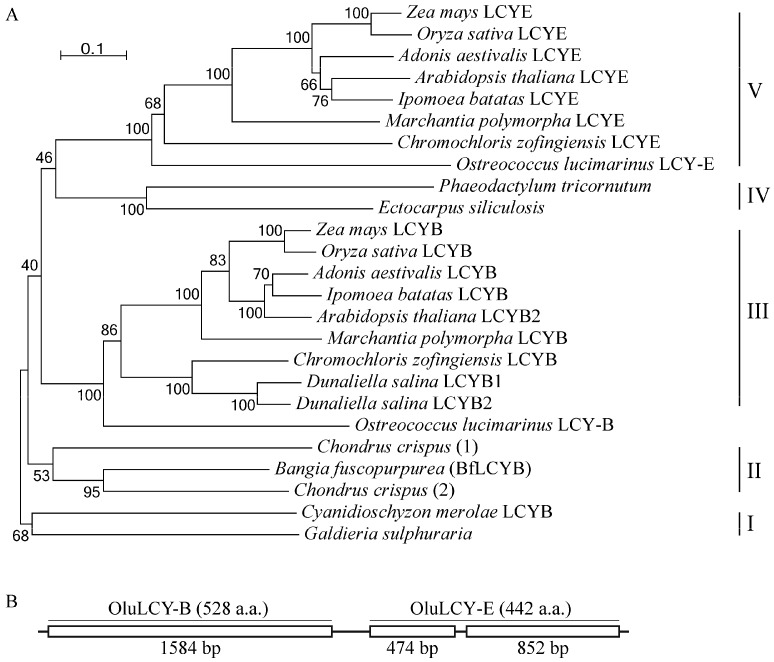
Phylogenetic analysis of plant LCYs. (**A**) Sequences of the functionally characterized LCYs from *Zea mays* (LCYB, NP_001169155.1; LCYE, NP_001146840.1), *Oryza sativa* (LCYB, XP_015627234.1; LCYE, XP_015622198.1), *Arabidopsis thaliana* (LCYB, NP_187634.1; LCYE, NP_200513.1), *Ipomoea batatas* (LCYB, AGL44392.1; LCYE, BAW34178.1), *Adonis aestivalis* (LCYB, AAK07430.1; LCYE, AAK07432.1), *Marchantia polymorpha* (LCYB, BAO27799.1; LCYE, BAO27800.1), *Chromochloris zofingiensis* (LCYB, CBH31263.1; LCYE, CCG06343.1), *Dunaliella salina* (LCYB1, ACA34344.1; LCYB2, ANY98896.1), *Ostreococcus lucimarinus* (LCYB, XP_001422489.1; LCYE, XP_001422490.1), *Bangia fuscopurpurea* (LCYB, KX943552) and *Cyanidioschyzon merolae* (XP_005536481.1), and their homologs from *Ectocarpus siliculosus* (CBN78004.1), *Phaeodactylum tricornutum* (XP_002176612.1), *Chondrus crispus* (1, XP_005713399.1; 2, XP_005715292.1) and *Galdieria sulphuraria* (XP_005708098.1) are analyzed using MEGA 6. Scale bar, 10% sequence divergence. (**B**) Structure of the *Ostreococcus lucimarinus LCY* gene that has two tandemly arranged regions for LCYB (OluLCY-B) and LCYE (OluLCY-E) activities.

Using sequences of functionally characterized LCYs, we identified LCY homologs in heterokonts and red algae of which full genomes have been sequenced. For heterokonts, in the genome of either *Ectocarpus siliculosus* (brown alga) or *Phaeodactylum tricornutum* (diatom), there is only a single gene encoding an LCY homolog (Figure 5A). However, for the multicellular red alga *Chondrus crispus*, our search identified two genes for putative LCYs in its genome (Figure 5A). Our phylogenetic analysis divided LCYs and their homologs into five major clades. Clade I comprises members from single-cell red algae, including the LCYB from *Cyanidioschyzon merolae* and a homolog from *Galdieria sulphuraria*. Clade II has members from multicellular red algae, including BfLCYB in this study and the two homologs from *Chondrus crispus*. Clade IV has LCY homologs from heterokonts. The other two clades, III and V, are LCYBs and LCYEs, respectively, from the green lineage (green algae and land plants) (Figure 5A).

A recently study by Blatt et al. [24] reported a gene (*OluLCY*) encoding a lycopene β-cyclase/lycopene ε-cyclase/light-harvesting complex-fusion protein from the green alga *Ostreococcus lucimarinus*. Our sequence analysis of this gene identified two coding regions (*OluLCY-B* and *OluLCY-E*) that are tandemly arranged, separated by a short intron (Figure 5B). In our phylogenetic tree, peptides encoded by these two regions are grouped with functionally characterized LCYBs and LCYEs, respectively (Figure 5B).

## 3. Discussion

In green lineage organisms (green algae and land plants), carotenoids with both β- and ε-rings are found, and separate LCYBs and LCYEs catalyzing the corresponding cyclization steps are also characterized from different species [1,25]. Our phylogenetic analysis clustered LCYBs and LCYEs from these organisms into separate clades (IV and V, Figure 5), showing a divergence of lycopene β- and ε-cyclase activities (Clades IV and V, Figure 5A) before the origination of the green lineage. This is also supported by the identification of the *OluLCY* gene, which has two tandemly arranged regions for peptides with LCYB and LCYE activities, respectively, suggesting probable gene duplication and divergence.

In red algae and heterokonts, although their carotenoid constituents have been studied, there is not sufficient information on their LCYs [2,26]. In both single-cell red algae and heterokonts, only β-carotene and its derivatives were found. Consistently, LCYs from *Cyanidioschyzon merolae* possess only β-cyclization activity, and studies in diatoms found no sequences showing similarity to *LCYE* genes either [16,26,27,28]. Therefore, it is reasonable to postulate that members of Clades I and IV in Figure 5A are all LCYBs.

For multicellular red algae, which accumulates carotenoids with both β- and ε-rings, studies on their LCYs are lacking. In this work, we studied lycopene cyclization in *Bangia fuscopurpurea*, which is considered to be one of the simplest macrophytic red algae. Our molecular cloning and functional characterization results demonstrated that *B. fuscopurpurea* has a functional LCYB (BfLCYB), which is capable of catalyzing β-cyclization at both ends of the acyclic lycopene or at either end of the monocyclic γ-carotene or δ-carotene. By scanning the genome sequence of another multicellular red alga, *Chondrus crispus*, we found two homolog genes for LCYs. These remind us of the possible existence of another as-yet-unidentified LCYE homolog responsible for ε-cyclization in *B. fuscopurpurea*. Cloning and functional characterization of this second LCY from *B. fuscopurpurea* should help to decipher carotenoid metabolism and its evolution in red algae.

## 4. Materials and Methods

### 4.1. Material and Growth Condition

Thalli of *Bangia fuscopurpurea* (#Bangia-MC1F, Jiangsu Institute of Oceanology and Marine Fisheries, Nantong, Jiangsu, China) were initially collected from cultivated populations in Putian, Fujian Province, China (24°59′ N, 118°48′ E) [29] and then subcultured in sterilized seawater in our laboratory. Ambient air filtered through a 0.45 μm filter was gently supplied to the culture. The growth conditions were 16 °C with a 12 h/12 h light/dark regime under 80 μmol m^−2^ s^−1^ irradiance. Seawater was changed every week.

### 4.2. Sequence Analysis

Sequences of functionally characterized LCYs from *Zea mays*, *Oryza sativa*, *Arabidopsis thaliana*, *Ipomoea batatas*, *Adonis aestivalis*, *Marchantia polymorpha*, *Chromochloris zofingiensis*, *Dunaliella salina*, *Ostreococcus lucimarinus* and *Cyanidioschyzon merolae* were downloaded from GenBank, and their homologs in *Ectocarpus siliculosus*, *Phaeodactylum tricornutum*, *Chondrus crispus* and *Galdieria sulphuraria* were searched in GenBank using the blastP algorithm with the sequences of known LCYs as queries. Sequence alignment was performed using ClustalW in MEGA 6 [30]. A bootstrap (1000 replicates) neighbor-joining phylogenetic tree was also generated using MEGA 6. 4.3. Molecular manipulation.

Total RNA was isolated from *Bangia fuscopurpurea* material as previously described [31] and treated with DNase (Promega, Madison, WI, USA) to eliminate any genomic DNA contamination. For rapid amplification of cDNA ends (RACE), the SMARTer RACE cDNA Amplification Kit (TaKaRa, Shiga, Japan) was used to synthesize a full-length cDNA pool.

According to the alignment of the functionally characterized LCYs, two pairs of degenerate primers (DF1/DR1, DF2/DR2, sequences of all primers used in this work are listed in Table 1) were designed from the conserved regions [32], and used for amplifying a corresponding fragment from the *B. fuscopurpurea* cDNA pool. After two rounds of nested PCR, amplified products were subcloned and sequenced by GenScript (Nanjing, China). The sequences of the cloned fragments were queried against GenBank using the tblastX algorithm to analyze their similarities to known or predicted *LCY* genes.

Using the sequences that had high similarity to known *LCY*s, three pairs of primers (RF1/RR1, RF2/RR2 and RF3/RR3) were designed for 5′- and 3′-RACEs, respectively, using the SMARTer RACE kit. After three rounds of nested PCR, two overlapping fragments were amplified from the cDNA pool, subcloned and sequenced. The sequences were assembled into a putative cDNA sequence that contained both translation initiation and stop codons. We then designed an additional pair of primers (HF and ER) from the assembled sequence to amplify the full-length ORF from the cDNA pool for confirming the existence of this transcript (named *BfLCYB*).

To study the genomic sequence of *BfLCYB*, genomic DNA was extracted according to Yang et al. [31]. Primers HF and ER were used to amplify its coding region. A Genome Walking Kit (TaKaRa) was used to amplify the 5′ and 3′ flanking sequences. All amplicons were purified, subcloned, sequenced and assembled. Two primers (gHF and gER) were used to amplify from the genomic DNA for confirming the assembled genomic DNA sequence of *BfLCYB*.

### 4.3. Subcellular Localization

Prediction of the subcellular localization of BfLCYB was performed using TargetP [19], YLoc [20] and ChloroP [21].

For studying the subcellular localization of BfLCYB, protoplasts isolated from *Arabidopsis thaliana* mesophyll were transiently transformed [22]. The full-length ORF of *BfLCYB* was amplified with primers HF-SpeI and ER-SpeI to incorporate restriction sites. The amplicon was purified, digested with SpeI, and subcloned into pA7-YFP (kindly provided by Dr. Hongquan Yang). The transformed protoplasts were cultured in 24-well plates at room temperature for 16 h in the dark. Fluorescence signal from the BfLCYB-YFP fusion protein was observed using a confocal laser scanning microscope (Olympus FluoView FV1000, Tokyo, Japan). The transient expression experiments were repeated independently at least three times.

### 4.4. Functional Characterization

To assess the catalytic activity of BfLCYB, we used the pigment complementation system [23]. Two vectors, pAC-LCY and pAC-EPSILON were provided by Dr. Cunningham. The full-length ORF of *BfLCYB* was amplified with primers HF-BamHI and ER-BamHI to incorporate restriction sites. The amplified product was subcloned into pMAL-C5X (New England BioLabs, Ipswich, MA, USA) to produce pMAL-BfLCYB. pMAL-BfLCYB was co-transformed with pAC-LCY, or pAC-ΕPSILON, into *E. coli* BL21(DE3) cells (New England BioLabs, Ipswich, MA, USA). After overnight growth at 37 °C on Luria-Bertani (LB), plants containing 34 μg mL^−1^ chloramphenicol and 100 μg mL^−1^ ampicillin, positive co-transformed single colonies were picked and used to inoculate 8 mL LB medium containing the same antibiotics. One mL of the overnight culture was transferred to 100 mL of the same medium and grown at 37 °C until reaching an OD600 of 0.30. Protein expression was induced by adding isopropyl β-thiogalactopyranoside (IPTG) to a final concentration of 50 μmol L^−1^. After growing for 16 h at 18 °C, bacterial cells were harvested by centrifugation and resuspended in 80% (*v*/*v*) acetone to extract the pigments.

### 4.5. Carotenoid Analysis

Separation of carotenoid components was performed by reverse-phase HPLC [33]. A Waters 2695 separation module and 2998 photodiode array detector (PDA) were used with a Spherisorb ODS2 column (5 μm, 4.6 mm × 250 mm, Waters, Milford, MA, USA). A 45 min gradient of ethyl acetate (0% to 100%) in acetonitrile-water-triethylamine (9:1:0.01, *v/v/v*) at a flow rate of 1 mL min^−1^ was used, and the absorbance of the eluent at 440 nm was monitored [34]. The identity of the carotenoid in each peak was further confirmed by their UV/visible spectra recorded by the PDA detector.

## Figures and Tables

**Table 1:** Primers used in this work.

Primer	Sequence (5′-3′) ^1,2^
DF1	MMNAAYTAYGGNKKNTGGBWNGAYGAR
DR1	GGRTGNACSADNSHNGCNGYNSCNCCNAWNSC
DF2	ATGBTNYTNATGGAYKDNMGNGA
DR2	RNVNNCCNCCNAYNGGDATNWV
RR1	GGCCCCCCCATGGGGATCAGACAAAACTC
RR2	CAGACAAAACTCCTCATCGAGCACCCGC
RR3	CACCCGCGTCACGGTGATGCCGTC
RF1	GGTGCTGATGGACTACCGGGATGG
RF2	GGGATGGGCACATGCAGGGGGAC
RF3	GGACGCCGCGGGGCGGGCCGAGTC
HF	ATGGAGGCGTTCATCCCCGCCTC
ER	CTACGCCTCGTCCTCTGGCAGCGG
gHF	AATCAGTCAGTTTCGGTGATCTTTC
gER	CCGCCGAAACGGCCTACCCCT
SpeI-LCY-F	GACTAGTATGGAGGCGTTCATCCCCGCCTC
SpeI-LCY-R	GGGACTAGTCTACGCCTCGTCCTCTGGCAGCGG
BamHI-LCY-F	GGATCCATGGAGGCGTTCATCCCCGCCTC
BamHI-LCY-R	GGGGATCCCTACGCCTCGTCCTCTGGCAGCGG

**^1^** Codes for degenerate nucleotides are M (A/C), R (A/G), W (A/T), S (C/G), Y (C/T), K (G/T), V (A/C/G), H (A/C/T), D (A/G/T), B (C/G/T) and N (any nucleotide); **^2^** Restriction sites are underlined.

## References

[1] Cazzonelli C.I., Pogson B.J. (2010). Source to sink: Regulation of carotenoid biosynthesis in plants. Trends Plant Sci..

[2] Takaichi S. (2011). Carotenoids in algae: Distributions, biosyntheses and functions. Mar. Drugs.

[3] Britton G., Liaaen-Jensen S., Pfander H. (2004). Carotenoids: Handbook.

[4] Cazzonelli C.I. (2011). Carotenoids in nature: Insights from plants and beyond. Funct. Plant Biol..

[5] Al-Babili S., Bouwmeester H.J. (2015). Strigolactones, a novel carotenoid-derived plant hormone. Annu. Rev. Plant Biol..

[6] Tian S.L., Li L., Shah S.N.M., Gong Z.H. (2015). The relationship between red fruit colour formation and key genes of capsanthin biosynthesis pathway in *Capsicum annuum*. Biol. Plant..

[7] Clotault J., Peltier D., Berruyer R., Thomas M., Briard M., Geoffriau E. (2008). Expression of carotenoid biosynthesis genes during carrot root development. J. Exp. Bot..

[8] Cunningham F.X., Gantt E. (2011). Elucidation of the pathway to astaxanthin in the flowers of *Adonis aestivalis*. Plant Cell.

[9] Ruiz-Sola M.Á., Rodríguez-Concepción M. (2012). Carotenoid biosynthesis in Arabidopsis: A colorful pathway. Arabidopsis Book.

[10] Sandmann G. (2002). Molecular evolution of carotenoid biosynthesis from bacteria to plants. Physiol. Plant..

[11] Cunningham F.X., Gantt E. (2001). One ring or two? Determination of ring number in carotenoids by lycopene ε-cyclases. Proc. Natl. Acad. Sci. USA.

[12] Harjes C.E., Rocheford T.R., Bai L., Brutnell T.P., Kandianis C.B., Sowinski S.G., Stapleton A.E., Vallabhaneni R., Williams M., Wurtzel E.T. (2008). Natural genetic variation in *Lycopene Epsilon Cyclase* tapped for maize biofortification. Science.

[13] Moise A.R., Al-Babili S., Wurtzel E.T. (2014). Mechanistic aspects of carotenoid biosynthesis. Chem. Rev..

[14] Römer S., Argyroudi-Akoyunoglou J.H., Senger H. (1999). Carotenoids in higher plants and algae. The Chloroplast: From Molecular Biology to Biotechnology.

[15] Schubert N., García-Mendoza E., Pacheco-Ruiz I. (2006). Carotenoid composition of marine red algae. J. Phycol..

[16] Takaichi S., Yokoyama A., Uchida H., Murakami A. (2016). Carotenogenesis diversification in phylogenetic lineages of Rhodophyta. J. Phycol..

[17] Cunningham F.X., Lee H., Gantt E. (2007). Carotenoid biosynthesis in the primitive red alga *Cyanidioschyzon merolae*. Eukaryot. Cell.

[18] Butterfield N.J. (2000). *Bangiomorpha pubescens* n. gen., n. sp.: Implications for the evolution of sex, multicellularity, and the Mesoproterozoic/Neoproterozoic radiation of eukaryotes. Paleobiology.

[19] Emanuelsson O., Nielsen H., Brunak S., von Heijne G. (2000). Predicting subcellular localization of proteins based on their N-terminal amino acid sequence. J. Mol. Biol..

[20] Briesemeister S., Rahnenfuhrer J., Kohlbacher O. (2010). YLoc—An interpretable web server for predicting subcellular localization. Nucleic Acids Res..

[21] Emanuelsson O., Nielsen H., Von Heijne G. (1999). ChloroP, a neural network-based method for predicting chloroplast transit peptides and their cleavage sites. Protein Sci..

[22] Yoo S.-D., Cho Y.-H., Sheen J. (2007). *Arabidopsis* mesophyll protoplasts: A versatile cell system for transient gene expression analysis. Nat. Protoc..

[23] Cunningham F.X., Gantt E. (2007). A portfolio of plasmids for identification and analysis of carotenoid pathway enzymes: *Adonis aestivalis* as a case study. Photosynth. Res..

[24] Blatt A., Bauch M.E., Porschke Y., Lohr M. (2015). A lycopene β-cyclase/lycopene ε-cyclase/light-harvesting complex-fusion protein from the green alga *Ostreococcus lucimarinus* can be modified to produce α-carotene and β-carotene at different ratios. Plant J..

[25] Lohr M., Im C.S., Grossman A.R. (2005). Genome-based examination of chlorophyll and carotenoid biosynthesis in *Chlamydomonas reinhardtii*. Plant Physiol..

[26] Bertrand M. (2010). Carotenoid biosynthesis in diatoms. Photosynth. Res..

[27] Coesel S., Oborník M., Varela J., Falciatore A., Bowler C. (2008). Evolutionary origins and functions of the carotenoid biosynthetic pathway in marine diatoms. PLoS ONE.

[28] Kuczynska P., Jemiola-Rzeminska M., Strzalka K. (2015). Photosynthetic pigments in diatoms. Mar. Drugs.

[29] Wang W.-J., Zhu J.-Y., Xu P., Xu J.-R., Lin X.-Z., Huang C.-K., Song W.-L., Peng G., Wang G.-C. (2008). Characterization of the life history of *Bangia fuscopurpurea* (Bangiaceae, Rhodophyta) in connection with its cultivation in China. Aquaculture.

[30] Tamura K., Stecher G., Peterson D., Filipski A., Kumar S. (2013). MEGA6: Molecular Evolutionary Genetics Analysis version 6.0. Mol. Biol. Evol..

[31] Yang L.-E., Jin Q.-P., Xiao Y., Xu P., Lu S. (2012). Improved methods for basic molecular manipulation of the red alga *Porphyra umbilicalis* (Rhodophyta: Bangiales). J. Appl. Phycol..

[32] Ramos A., Coesel S., Marques A., Rodrigues M., Baumgartner A., Noronha J., Rauter A., Brenig B., Varela J. (2008). Isolation and characterization of a stress-inducible *Dunaliella salina Lcy-β* gene encoding a functional lycopene β-cyclase. Appl. Microbiol. Biotechnol..

[33] Yang L.-E., Huang X.-Q., Hang Y., Deng Y.-Y., Lu Q.-Q., Lu S. (2014). The P450-type carotene hydroxylase PuCHY1 from *Porphyra* suggests the evolution of carotenoid metabolism in red algae. J. Integr. Plant Biol..

[34] Norris S.R., Barrette T.R., DellaPenna D. (1995). Genetic dissection of carotenoid synthesis in Arabidopsis defines plastoquinone as an essential component of phytoene desaturation. Plant Cell.

